# Assistance of metagenomics next-generation sequencing for diagnosis of adenovirus pericarditis with pericardial effusion in a child: a case report and literature review

**DOI:** 10.3389/fped.2023.1174326

**Published:** 2023-06-12

**Authors:** Liangkang Lin, Min Xu, Haiyang Zhang

**Affiliations:** Department of Pediatric Intensive Care Unit, West China Second University Hospital, Sichuan University, Key Laboratory of Birth Defects and Related Diseases of Women and Children (Sichuan University), Ministry of Education, Chengdu, China

**Keywords:** adenovirus, pericardial effusion, pericarditis, metagenomics next-generation sequencing, case report

## Abstract

Human adenoviruses (HAdVs) can cause infection at any age but are most common in the pediatric population, especially young children and infants, with a peak incidence in infants and children from 6 months to 5 years of age. Adenovirus infection can cause severe pneumonia, but pericarditis from adenovirus infection was rare. This article reports a case of a 2-year-old patient with pericarditis caused by adenovirus infection and a moderate pericardial effusion. We detected positive adenovirus nucleic acid in the patient’s blood by polymerase chain reaction assay. In addition, HAdVs were identified by metagenomics next-generation sequencing (mNGS) in blood and pericardial effusion. According to the test results and clinical practice, active symptomatic and supportive treatment was given, and finally the child recovered and was discharged from the hospital. Comprehensive and accurate diagnosis of pathogens is a prerequisite for effective treatment, and mNGS provides an effective means for diagnosing rare adenovirus myocarditis in children.

## Introduction

Human adenovirus (HAdV), a member of the *Adenoviridae* family, is a medium-sized (70–100 nm) non-enveloped virus with an icosahedral nucleocapsid containing a double-stranded linear DNA genome of 34–36 kbp in length (1). HAdV is a common pathogen of respiratory tract infection in children. Recently, adenovirus has also been reported as a pathogen of viral myocarditis in children, but the occurrence of such myocarditis is rare (1). Although HAdV infection can lead to myocarditis in children, it is extremely dangerous to cause pericarditis with moderate-to-massive pericardial effusion, which usually progresses rapidly and is difficult to detect early, eventually leading to death.

In this case, we reported a rare case of pericarditis due to HAdV infection in a 2-year-old patient. The initial treatment was unsatisfactory, and echocardiography revealed moderate-to-massive pericardial effusion. The patient then presented with cardiac tamponade. His condition improved after pericardiocentesis. After that, adenovirus infection was confirmed by polymerase chain reaction (PCR) and metagenomics next-generation sequencing (mNGS), and the treatment was accordingly adjusted in time. The patient's condition improved, and he was discharged from the hospital. Compared with traditional microbial diagnostic methods, mNGS can achieve early detection and rapid diagnosis, and it will be promising for future pathogen diagnosis, offering unbiased and hypothesis-free properties that makes it a universal pathogen detection method (2).

## Case presentation

A 2-year-old male patient was admitted to the Pediatric Intensive Care Unit (PICU) of West China Second University on 10 May 2022. On admission, his chief complaint was fever and listlessness, occasional non-projectile vomiting with anorexia, and shortness of breath. Due to acute onset and rapid progression of the disease, the patient was given oxygen inhalation, empirical ceftriaxone anti-infection (80 mg/kg q.d. for 1 day), and nebulization in pre-hospital emergency center, within the critical symptoms did not ease. Afterward, he developed low blood pressure, increased heart rate, and progressive increased shortness of breath and required assisted ventilation. The patient was then transferred to the PICU for advanced treatment. The child's birth history, personal history, and family history were normal, and there was no history of recurrent respiratory infections and congenital or acquired heart disease.

**Figure 1:**
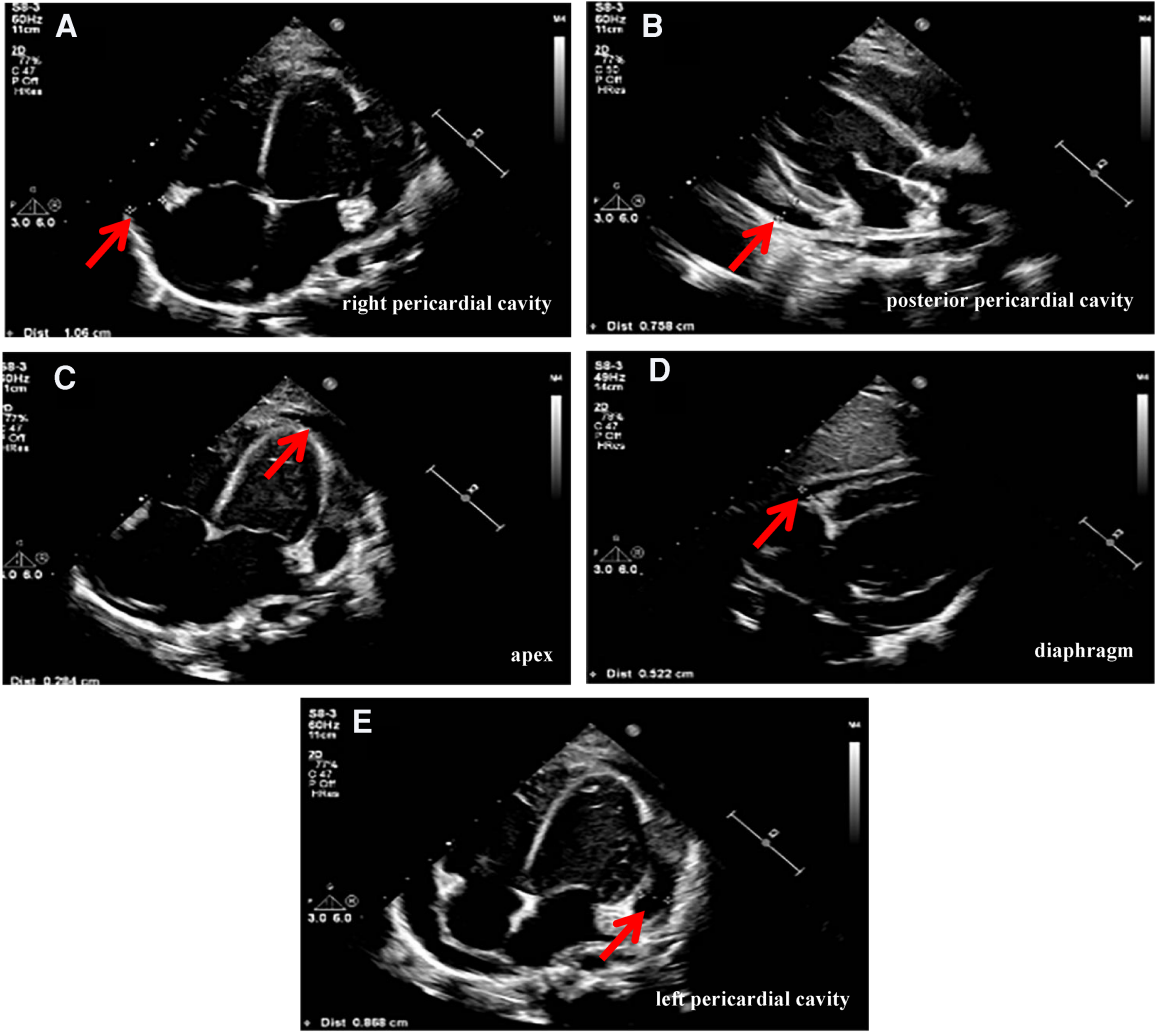
Echocardiography (on admission) showed pericardial effusion: (**A**) posterior pericardial cavity of 7.6 mm. (**B**) Left pericardial cavity of 8.7 mm. (**C**) Right pericardial cavity of 10.6 mm. (**D**) Apex of 2.8 mm. (**E**) Diaphragm of 5.2 mm.

**Figure 2:**
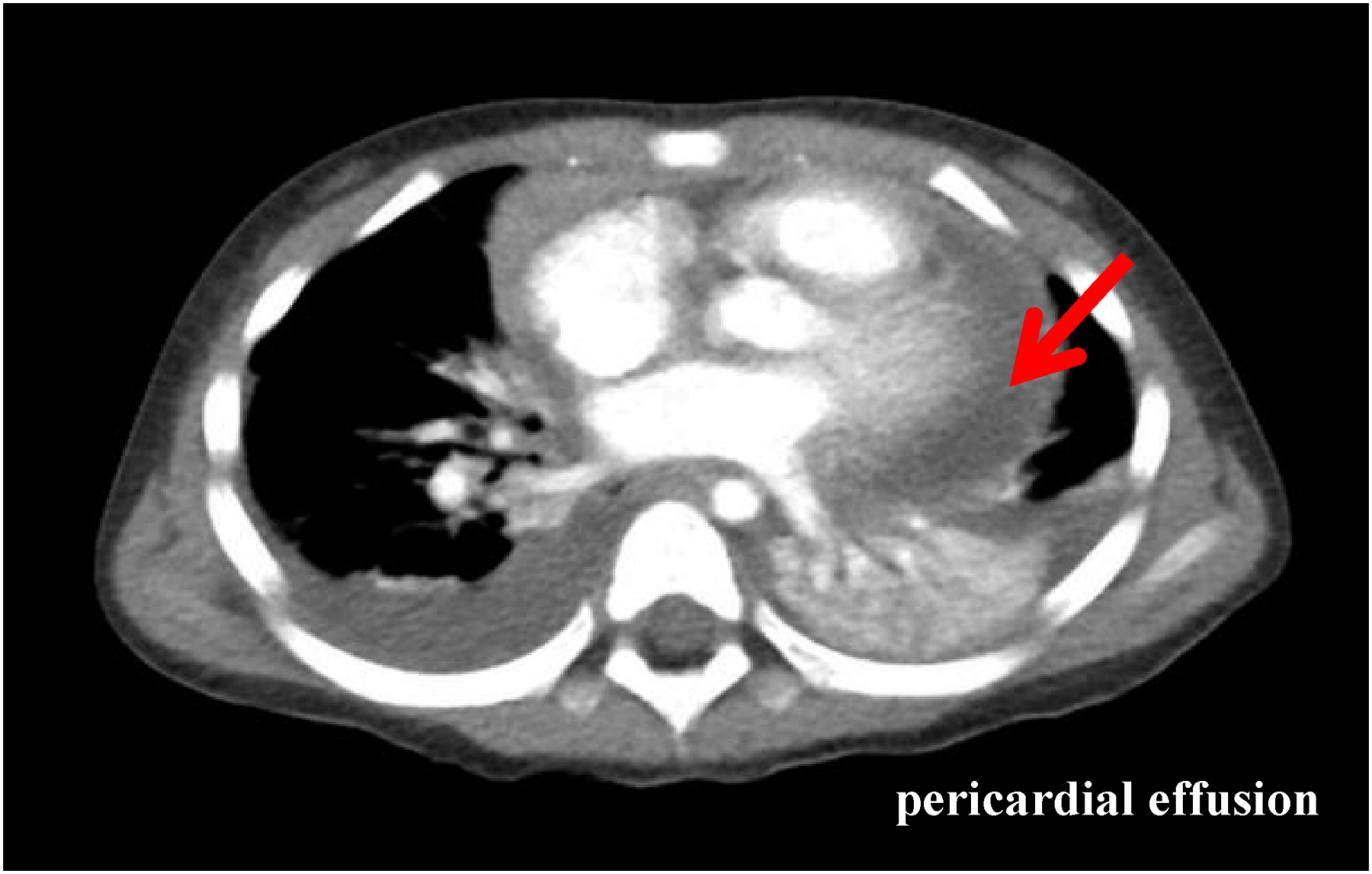
Enhanced chest CT (the third day of admission): bilateral lung infection with massive consolidation; enlarged heart, moderate pericardial effusion, pericardial thickening, and enhancement; and a small amount of bilateral pleural effusion.

Physical examination data at admission showed temperature of 36.5°C, respiratory rate of 35 times/min, pulse rate of 141 times/min, blood pressure of 95/52 mmHg, Glasgow Coma Score (GCS) of 13 (E4M5V4), and weight of 15 kg. White secretions were observed on the left buccal mucosa, which was not easy to wipe off. Three-concave sign was negative, the breath sounds of both lungs were rough with a few small moist rales, and no stridor was heard. The heart sounds were slightly low, blunt, and distant, the rhythm was normal, and no murmurs were heard in the valve areas. Routine blood examination showed white blood cell (WBC) count of 16.8 × 10^9^/L, blood platelet (PLT) count of 256 × 10^9^/L, hemoglobin (Hb) of 108 g/L, C reactive protein (CRP) of 109.4 mg/L, and procalcitonin of 0.87 ng/ml. Myocardial markers test showed troponin I (cTnI) of 0.207 μg/L and B-type natriuretic peptide (BNP) of 475.1 pg/ml. Coagulation function showed D-dimer of 2.59 mg/L and fibrinogen degradation product (FDP) of 6.40 μg/ml. Liver and kidney function tests and serum electrolytes were all normal. The electrocardiogram indicated changes in T wave. Echocardiography showed ejection fraction (EF) of 71%, fraction shortening (FS) of 38%, and moderate pericardial effusion (posterior pericardial cavity of 7.6 mm, left pericardial cavity of 8.7 mm, right pericardial cavity of 10.6 mm, apex of 2.8 mm, diaphragm of 5.2 mm, Figure 1). Chest x-ray showed double pneumonia, and the cardiac shadow was significantly enlarged. Enhanced chest computed tomography (CT) showed bilateral lung infection, enlarged heart, moderate pericardial effusion, pericardial thickening and enhancement, and a small amount of bilateral pleural effusion (Figure 2).

**Figure 3:**
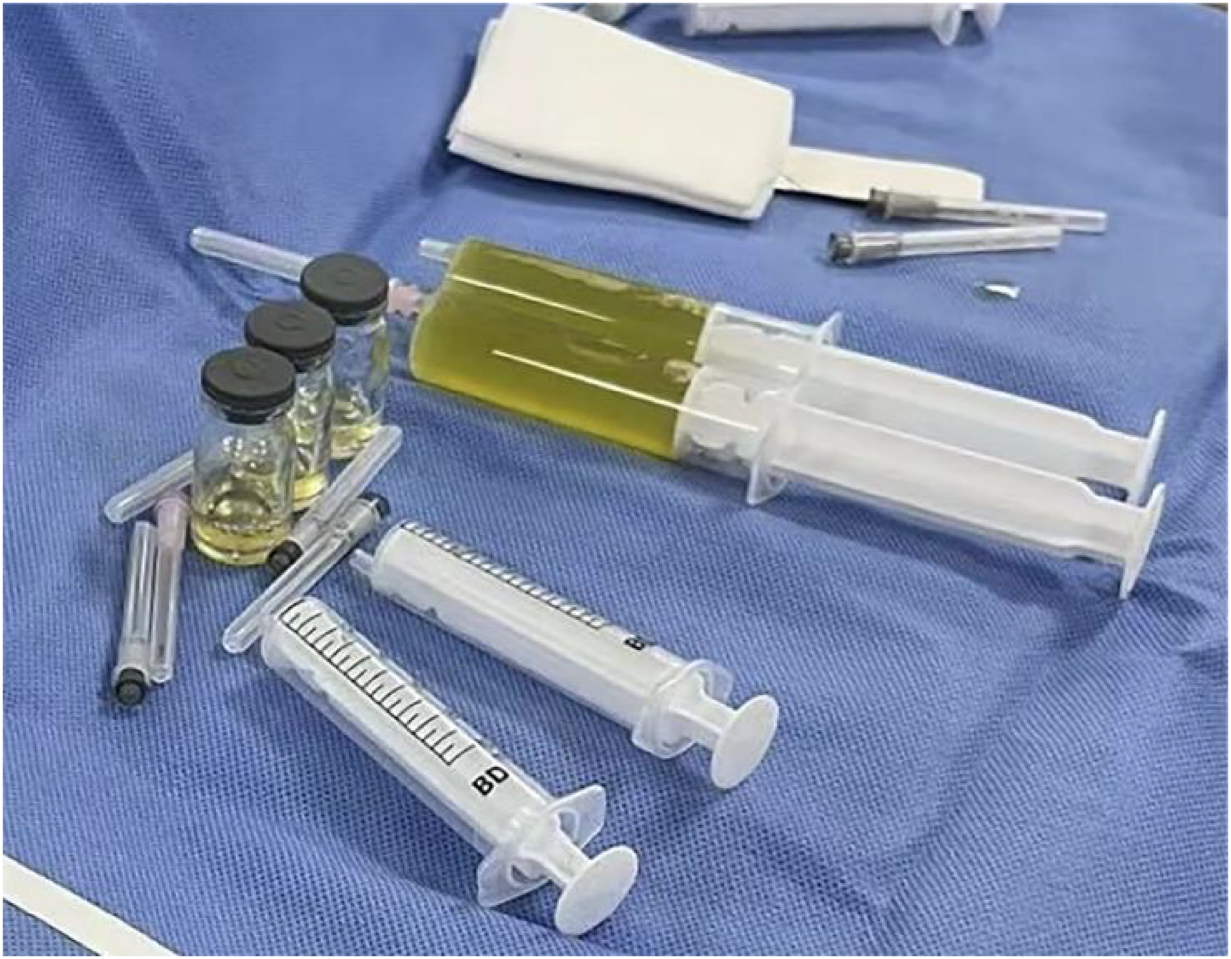
Ultrasound-guided pericardiocentesis was performed after bedside local anesthesia. Pericardiocentesis extracted 180 ml of pale yellow, clear, non-coagulated pericardial effusion.

**Figure 4:**
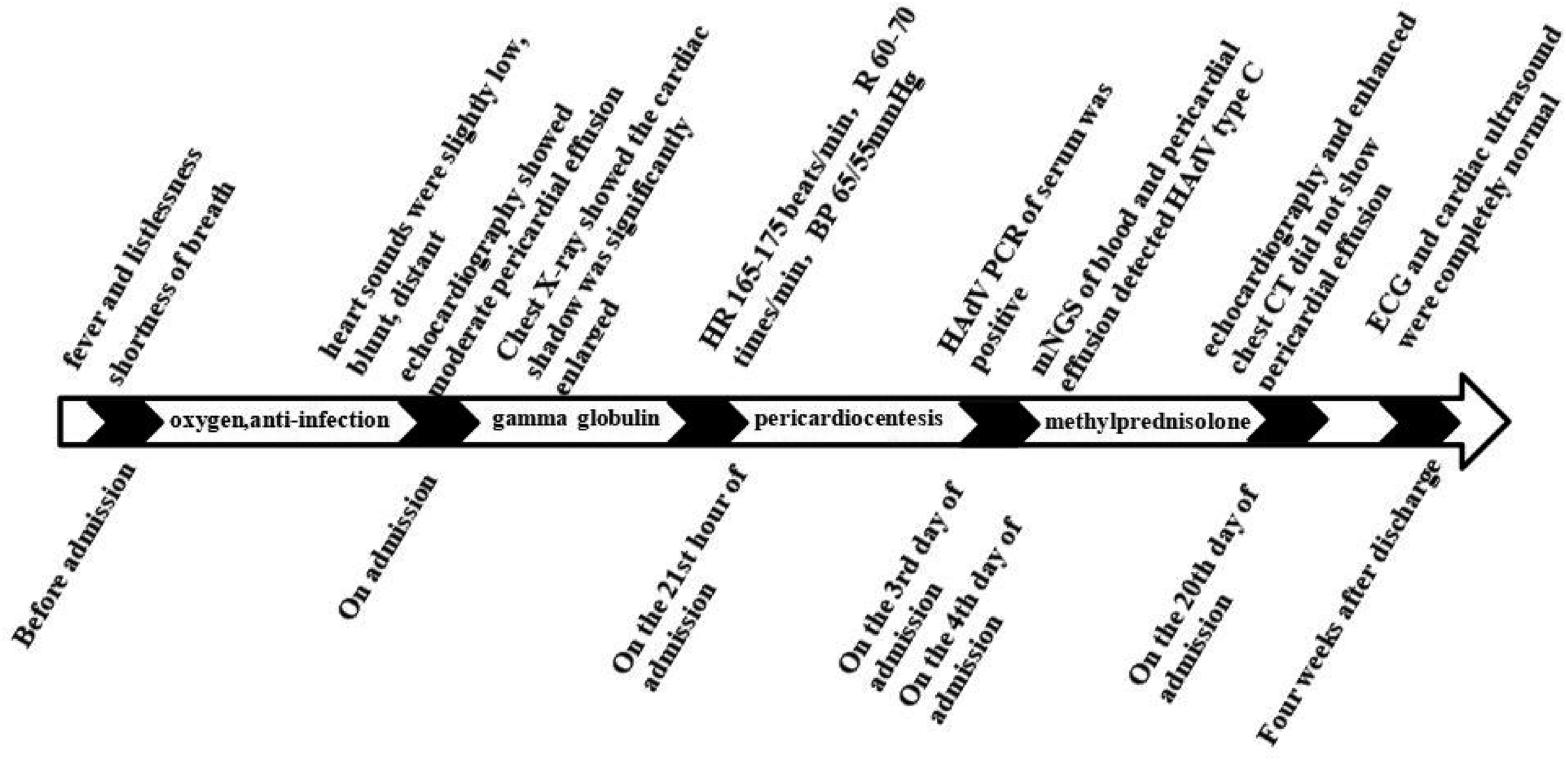
The timeline of patient treatment and disease progression.

The patient was preliminarily diagnosed with sepsis, pericardial effusion, severe pneumonia with coagulopathy, thrush, and myocardial injury. He was timely given intravenous infusion of ceftriaxone combined with vancomycin for anti-infection, gamma globulin (1 g/kg for 1 day) pulse therapy for anti-inflammatory, creatine phosphate (65 mg/kg q.d. for 5 days), and vitamin C (100 mg/kg b.i.d. for 5 days) for protection against myocardial ischemia. On the 21st hour of admission, the patient’s blood pressure progressively decreased. The heart rate fluctuated at 165–175 beats/min, and the respiratory fluctuation was 60–70 times/min. At that time, the peripheral blood pressure showed 65/55 mmHg, and the pulse pressure difference was only about 10 mmHg, indicating the presence of pericardial tamponade. Physical examination at that time suggested distant heart sounds and moderate-massive moist rales in both lungs. Bedside ultrasound-guided pericardiocentesis was performed upon admission (local anesthesia with lidocaine was administered). The puncture point was 1 cm outside the border of cardiac dullness at the apex of the fifth intercostal space. The needle was inserted 3 cm in the direction of the right scapular region, and 180 ml of pale yellow, clear, non-coagulated pericardial effusion was extracted (Figure 3). We extracted 10 ml of pericardial effusion for mNGS, pathology, routine, and biochemical tests. At the same time, 4 ml of peripheral blood sample was extracted for mNGS. Approximately 10 min after pericardiocentesis, the heart rate of the patient decreased, fluctuating at 120–135 beats/min, and the blood pressure increased to 95/58 mmHg (normal pulse pressure reappeared). On the second day of admission, *Mycoplasma pneumoniae* and *Chlamydia pneumoniae* PCR of serum were negative, and serum G [(1–3)-β-d-glucan] test and GM (galactomannan) test were negative. On the third day of admission, HAdV PCR of serum was positive, and cytokines showed interleukin-1β of 6.84 pg/ml, interleukin-2 receptor of 811.00 U/ml, interleukin-6 of 76.60 pg/ml, interleukin-8 of 7.02 pg/ml, interleukin-10 of 17.90 pg/ml, and tumor necrosis factor-alpha of 17.90 pg/ml. A pericardial effusion examination showed a nucleated cell count of 2,033 × 10^6^/L (neutrophil granulocytes of 77.0%, lymphocytes of 3.0%, monocytes of 20.0%). The total protein of pericardial effusion is 52.2 g/L, glucose is 4.70 mmol/L, lactate dehydrogenase (LDH) is 1,145.0 U/L, and adenosine deaminase (ADA) is 32.2 U/L. These results suggested that the pericardial effusion was inflammatory exudate. Coxsackie virus antibody, rheumatism screening, autoantibody, and tumor marker examination showed no obvious abnormality. Echocardiography showed a small amount of pericardial effusion, and left ventricular systolic function was normal. The electrocardiogram test result was normal. According to the test results and the clinical symptoms of the child, the diagnosis was sepsis, severe pneumonia, and adenovirus infection with moderate pericardial effusion. On the fourth day of admission, mNGS of blood and pericardial effusion detected human mammalian adenovirus type C with 102 specific sequences (covering 52.34% of the nucleotide sequences) and 88 specific sequences (covering 48.17% of the nucleotide sequences), respectively. Moreover, the pathological result of pericardial effusion was negative. Based on the known test results, we stopped the use of antibiotics and added methylprednisolone (1 mg/kg q.d. for 5 days) to reduce inflammation. The vital signs of the child were basically normal under non-invasive assisted ventilation, and the ventilator parameters were adjusted down and gradually transitioned to nasal cannula for oxygen inhalation. On the sixth day of admission, the child stopped oxygen inhalation, and the intravenous infusion of methylprednisolone was changed to oral prednisone acetate (5 mg b.i.d. for 14 days). On the 20th day of admission, HAdV PCR of serum we retested was negative, and echocardiography and enhanced chest CT did not show pericardial effusion. The patient was discharged with perfectly normal vital signs. During the outpatient follow-up period of 4 weeks after discharge, the patient only took coenzyme Q10 orally on a regular basis. Physical examination, blood routine, electrocardiogram, and cardiac ultrasound were also completely normal during follow-up. Whole exome sequencing (WES) of this patient and his immediate family members up to three generations also showed no immunodeficiency-related mutations. We have summarized the timeline of patient treatment and disease progression in Figure 4.

## Systematic review

We searched in PubMed, Web of Science databases, Embase, and Medline from 1970 to 2023. The keywords used were “adenovirus,” “myocarditis,” and “pericarditis.” We extracted the following details of each article: first author, publication date, country, number of patients, age, symptoms, laboratory methods, diagnosis, treatment, and prognosis. We identified a total of four articles through our literature search, and characteristics of the cases are shown in Table 1 (3–6).

**Table 1:** The reported cases of myocarditis and pericarditis were caused by adenovirus.

Author	Country, year	Age	Male/female	Manifestation	Chest image	Sample	Method	Co-infection	Mechanical ventilation	Clinical diagnosis	Treatment	Outcome	Fundamental disease
Henson et al. (3)	The United States, 1971	11 months	0/1	Cyanotic, irritable, lethargic	Diffusely enlarged heart	Lung tissue	Cytopathic effect, antigen–antibody reaction	No	Yes	Respiratory failure, myocarditis	Oxygen, supplemental	Death	None
Treacy et al. (4)	Ireland, 2010	11 years	1/0	Sore throat	None	Myocardial tissue	PCR	No	No	Viral myocarditis	Cardiopulmonary resuscitation	Death	None
Valdés et al. (5)	Cuba, 2008	5months–15 years	4/2	Fever, anorexia, headache, oliguria	Slight cardiomegaly	Myocardial tissue	PCR	Not mentioned	Not mentioned	Viral myocarditis	Not mentioned	Death	Dilated cardiomyopathy, splenectomy, B-thalassemia, sick cell anemia
Briassoulis et al. (6)	Greece, 2000	4.5 years	0/1	Drowsiness, headache, vomiting	Cardiomegaly	Blood	PCR	No	Yes	Viral myocarditis	Mechanical ventilation, diuresis, fluid restriction, inotropic support with dobutamine	Survival	None

PCR, polymerase chain reaction.

### mNGS process and bioinformatic analysis

Approximately 200 µl of pericardial puncture fluid and peripheral blood samples were centrifuged at room temperature for 5,000 r/min for 10 min to obtain the supernatant. DNA in the supernatant was extracted and purified using the Tiangen nucleic acid extraction kit. Negative controls without any microorganisms and positive controls with mixed bacteria were set for each batch of samples. Qubit 2.0 (Thermo Fisher, United States) and agarose gel electrophoresis were used for the quality control of DNA extraction. According to the operating manual of Illumina DNA library construction kit (Illumina), known sequence fragments that can be identified with sequencing instruments were added at both ends of the DNA fragment to be tested for amplification and enrichment. Library quality control was completed by using Agilent 2,100 Bioanalyzer (Agilent, United States). A 75 bp single-ended high-throughput sequencing was performed on Illumina second-generation sequencer Pin NextSeq 550Dx.

fastp software was used to remove low-quality and redundant fragments and joint sequences from the original data to obtain high-quality data. Human hosts were removed by Bowtie2 comparison of human reference genome database. The reference used for human genome removal was Genome Reference Consortium human genome (build 38). The positive criteria for the detection of mNGS: In general, the pericardial effusion and blood of normal people were considered to be absolutely sterile, the detection of microorganisms was considered as pathogen detection, and the number of detected sequences of ≥3 is considered positive.

## Discussion

The incidence of HAdV infection peaks in infants and children aged 6 months to 5 years. Respiratory infections are the most common disease caused by HAdV in children. Different types of HAdV appear to exhibit different pathogenic properties and cause different diseases, suggesting differences in tissue tropism between HAdVs (7). Pericardial effusion caused by HAdV infection is rarely reported, and no similar case has been reported in China. The patient's condition progressed rapidly, and even cardiac tamponade occurred.

The pathophysiological mechanism of cardiac inflammation induced by viral infection has been an important area of research. Infectious pericarditis is usually the result of a combination of myocardial inflammatory cell infiltration and changes in cell/tissue osmotic pressure. Pericarditis caused by HAdV infection is not a simple myocardial cell inflammation. There are multiple cells in the heart involved in this complex process, such as vascular endothelial cells, monocytes, and fibroblasts (8). Direct damage by the virus and recognition of the virus by immune cells result in cardiomyocyte lysis and necrosis. Cytokine storm ensues, during which IL-1, IL-6, tumor necrosis factor-alpha (TNF-α), and type I and type II interferons (IFN) are released in large quantities, with sustained release of cells factors can cause myocarditis and pericarditis (9, 10). Pericardial effusion may be a secondary response to myocarditis and pericardial inflammation. The coxsackievirus and adenovirus receptor (CAR), a 46-kDa transmembrane protein, are the major receptors for adenoviruses. They are involved in cellular communication, facilitating viral binding and endocytosis. CAR is ubiquitously expressed in tight junctions in epithelial tissues, which are important barriers to prevent the free diffusion of solvents and proteins between the endothelial and epithelial cell layers. The expression of CAR is related to IL-6 (11, 12). Recent research has found that CAR leads to the development of cardiac inflammation independent of viral infection itself through inflammatory mitogen-activated protein kinase (MAPK) cascade signaling (13). At the same time, what had to be considered in our case was the oxidative stress and myocardial injury caused by increased myocardial oxygen demand due to severe hypoxia from acute respiratory injury. As pulmonary capillaries continue to spasm, hypoxia leads to pulmonary hypertension, a process that may lead to cardiac ejection dysfunction and increased capillary hydrostatic pressure, ultimately leading to pericardial effusion. However, not all patients have the same clinical outcome, which is determined by the interplay between pathogen virulence, host response, and therapeutic intervention. In addition, matrix metalloproteinases (MMPs) also deserve our attention; although not part of the immune system, they play a key role in the inflammatory response, including matrix degradation and promotion of leukocyte migration, as well as cytokine processing and regulation. MMPs are upregulated during viral myocarditis, often causing myocardial injury and dysfunction with higher cellular permeability, leading to the production of pericardial effusion (14).The clinical symptoms of cardiotoxic infections are not specific, and diagnosis is often based on the isolation of viruses from different biological samples. However, traditional methods for detecting viral antigens or virus culture have low sensitivity and are time-consuming for virus culture. When diagnosing pericarditis, it is hard to confirm the infection of the heart by the virus through the detection of serum antibodies or PCR. Endomyocardial biopsy (EMB) is the gold standard for the diagnosis of cardiac disease but involves greater risks, especially in pediatric or critically ill children. EMB is inappropriate for pediatric pericarditis, and many centers have abandoned EMB as a diagnostic tool. According to current guidelines, myocardial biopsy is not recommended for routine identification of causative viruses, as it does not affect treatment decisions and prognosis (15). HAdV is a double-stranded, linear DNA virus with genome sizes ranging from 34 to 37 kb, and all HAdVs have a similar genome organization. Molecular evolution of HAdVs through homologous recombination can lead to the emergence of new viruses that exhibit different tissue tropisms. To date, the types of HAdV are still increasing (16, 17), HAdV types 1–51 are characterized by serotyping, while the remaining types identified since 2007 are described by genomic and bioinformatic analyses. In previous studies, most nucleic acid detection methods for HAdV were designed based on hexon or fiber genes. However, due to the high diversity of adenovirus sequences, primers usually contain degenerate bases or use other strategies to overcome mismatched bases, and it is usually impossible to identify adenovirus subtypes, viral loads, and low copy number samples and often produces negative results (18). As noted above, the range of HAdV types has been expanding, so there is a necessity to expand detection methods to facilitate coverage of the entire human adenovirus spectrum. By directly sequencing the DNA or RNA in the sample, mNGS effectively overcomes the shortcomings of traditional detection methods, has extremely high specificity and sensitivity (19, 20), and can type HAdV, which is beneficial to our choice of treatment strategy. The patient in this case was positive for adenovirus nucleic acid in blood by PCR. At the same time, because of the existence of pericardial effusion, we conducted mNGS detection on the initial drainage fluid of pericardiocentesis and drainage. This corroborated with the positive result of serum adenovirus PCR, which helped us confirm the infection of the heart by adenovirus. Due to the extremely high sensitivity of mNGS, it is important to distinguish between true-positive and false-positive pathogens when interpreting mNGS results. Different from sputum and blood, pericardial effusion is a sterile body fluid, so when a positive pathogen is detected, even if the viral load/sequence number is small, we still consider it to be indicative of disease.

Treacy et al. once reported the first cases of sudden death from myocarditis caused by adenovirus serotype 3 in children and fatal cases of myocarditis caused by adenovirus subgenus C in children, respectively (4, 5). Consistent with these reports, our case presented with clinical manifestations such as fever, dyspnea, and increased heart rate, and the disease progressed rapidly. However, in contrast to the aforementioned cases, which were often diagnosed through serum PCR and postmortem cardiac tissue biopsy, we utilized mNGS to identify the pathogen, enabling a definitive diagnosis and subsequent treatment. The European Society of Cardiology (ESC) guidelines recommend the use of anti-inflammatory drugs for acute pericarditis, such as non-steroidal anti-inflammatory drugs (NSAIDs) and colchicine. Corticosteroids as second-line therapy for patients who are unresponsive or intolerant to NSAIDs and colchicine (15). Glucocorticoids are second-line therapy for patients who are unresponsive or intolerant to NSAIDs and colchicine. (21). Considering that the patient's pericardial effusion is not related to systemic inflammatory diseases, we did not choose aspirin, NSAIDs, or colchicine for treatment. Othman and Eldadah reported a case of intrapericardial corticosteroid injection (22). The patient's pericardial effusion completely resolved after injection and did not recur after intrapericardial steroid administration, which may be a new direction for the treatment of pericardial effusion in the future. However, in patients with massive pericardial effusion and cardiac tamponade, prompt pericardiocentesis is the first life-saving measure. In addition to this, to reduce the possible evolution to constrictive pericarditis and cardiomyopathy, underlying pathogenic mechanisms, such as viral infection or persistent and autoimmune-mediated myocardial injury, should be addressed. The immunosuppressive treatment of viral myopericarditis remains controversial, with some suggesting that immunosuppression may be beneficial in patients with systemic disease-related or autoimmune myocarditis but may increase viral replication and exacerbate myocardial injury in viral myocarditis (23, 24). There is currently a lack of worldwide guidelines to support such treatment options. However, there are no clinical studies to show whether patients will respond to this treatment. It is worth noting that any type of pericarditis has the potential to recur. As for children, pericardial disease may lead to impaired cardiac development, which must be taken seriously. Regular and dynamic follow-up echocardiography is necessary.

It is extremely rare for HAdV to cause moderate-to-massive pericardial effusion in children, and this is the first reported case in China. Pericarditis caused by HAdV infection progresses rapidly, so early identification and treatment are extremely important. The good sensitivity and specificity of mNGS in the detection of HAdV in pericardial effusion are satisfactory, which can help us identify specific pathogens well. Although major advances in the diagnosis and treatment of acute pericarditis are currently being made, there is still room for further research.

## Conclusion

The good sensitivity and specificity of mNGS in the detection of HAdV in pericardial effusion are satisfactory, which can help us accurately identify the rare critical disease in children. Although major advances in the diagnosis and treatment of acute pericarditis are currently being made, there is still room for further research.

## Data Availability

The datasets presented in this article are not readily available because of ethical and privacy restrictions. Requests to access the datasets should be directed to the corresponding author.
